# *Burkholderia cepacia* complex outbreak linked to a no-rinse cleansing foam product, United States – 2017–2018

**DOI:** 10.1017/S0950268822000668

**Published:** 2022-08-04

**Authors:** Sharon L. Seelman, Michael C. Bazaco, Allison Wellman, Cerisé Hardy, Marianne K. Fatica, Mei-Chiung Jo Huang, Anna-Marie Brown, Kimberly Garner, William C. Yang, Carla Norris, Heather Moulton-Meissner, Julie Paoline, Cara Bicking Kinsey, Janice J. Kim, Moon Kim, Dawn Terashita, Jason Mehr, Alvin J. Crosby, Stelios Viazis, Matthew B. Crist

**Affiliations:** 1 U.S. Food and Drug Administration, Center for Food Safety and Applied Nutrition, College Park, MD, USA; 2 U.S. Food and Drug Administration, Office of Emergency Operations, Silver Spring, MD, USA; 3 U.S. Food and Drug Administration, Center for Drug Evaluation Research, Silver Spring, MD, USA; 4 Division of Healthcare Quality Promotion, Centers for Disease Control and Prevention, Atlanta, GA, USA; 5 Pennsylvania Department of Health, Bureau of Epidemiology, Harrisburg, PA, USA; 6 California Department of Public Health, Richmond, CA, USA; 7 Los Angeles County Department of Public Health, Los Angeles, CA, USA; 8 New Jersey Department of Health, Trenton, NJ, USA

**Keywords:** *Burkholderia cepacia* complex, cleansing foam, outbreak investigation

## Abstract

In March 2018, the US Food and Drug Administration (FDA), US Centers for Disease Control and Prevention, California Department of Public Health, Los Angeles County Department of Public Health and Pennsylvania Department of Health initiated an investigation of an outbreak of *Burkholderia cepacia* complex (*Bcc*) infections. Sixty infections were identified in California, New Jersey, Pennsylvania, Maine, Nevada and Ohio. The infections were linked to a no-rinse cleansing foam product (NRCFP), produced by Manufacturer A, used for skin care of patients in healthcare settings. FDA inspected Manufacturer A's production facility (manufacturing site of over-the-counter drugs and cosmetics), reviewed production records and collected product and environmental samples for analysis. FDA's inspection found poor manufacturing practices. Analysis by pulsed-field gel electrophoresis confirmed a match between NRCFP samples and clinical isolates. Manufacturer A conducted extensive recalls, FDA issued a warning letter citing the manufacturer's inadequate manufacturing practices, and federal, state and local partners issued public communications to advise patients, pharmacies, other healthcare providers and healthcare facilities to stop using the recalled NRCFP. This investigation highlighted the importance of following appropriate manufacturing practices to minimize microbial contamination of cosmetic products, especially if intended for use in healthcare settings.

## Introduction

The *Burkholderia cepacia* complex (*Bcc*) initially emerged in the 1980s as opportunistic human pathogens causing severe and sometimes life-threatening infections in patients with cystic fibrosis [1]. Initially classified as *Pseudomonas cepacian*, it was initially thought to be one specific bacterial species, however *Bcc* now constitutes 24 closely related *Burkholderia* species that can cause opportunistic infections in humans [2]. These bacteria are ubiquitous in the environment, especially in soil and water, and survive with minimal nutritional requirements [3]. *Bcc* display intrinsic resistance to antimicrobial agents and are responsible for cepacia syndrome, which is a frequently lethal necrotising pneumonia accompanied by septicaemia, primarily in patients with cystic fibrosis [4]. In addition to the threat posed to patients with cystic fibrosis, there have been outbreaks of *Bcc* infections among both immunocompromised (such as HIV-positive individuals and cancer patients undergoing chemotherapy) and immunocompetent patients without cystic fibrosis [2, 5]. These infections have been linked to intrinsic or extrinsic contamination of various products used in healthcare settings, such as balloon pumps, commercially available washing gloves, ultrasound gels, nebulised or intravenous solutions, mouthwashes, prefabricated wet wipes or washcloths, antiseptics and disinfectant solutions [3, 6].

On 12 February 2018, the Pennsylvania Department of Health (PADOH) notified the US Centers for Disease Control and Prevention (CDC) of six cases of *Bcc* infections among patients from an acute care hospital (ACH). Then, on 7 March 2018, the California Department of Public Health (CDPH) notified CDC of eight cases of *Bcc* infections among patients from a cardiac care unit in an ACH. Follow-up with the state and local health departments led to concern that the clusters could be related and potentially linked to a common product. On 13 March 2018, the Pennsylvania ACH notified PADOH that a culture of a contaminated no-rinse cleansing foam product (NRCFP), produced by Manufacturer A, performed in their laboratory yielded *Bcc*. CDPH and Los Angeles County Public Health (LACPH) identified the California ACH also used the NRCFP of interest.

The NRCFP was a US Food and Drug Administration (FDA) regulated, commercially available, cosmetic product used for skin and perineal care in hospitals and other healthcare settings for people who are unable to shower or bathe for medical reasons. At the time of this investigation Manufacturer A was comprised of two separate legal entities that are referred to as a single entity for the purposes of this paper. On 16 March 2018, CDC notified FDA of the two clusters of *Bcc* infections. FDA worked with federal, state and local partners to conduct a joint outbreak investigation.

## Methods

### Epidemiologic investigation

After the initial notifications, PADOH, CDPH and LACPH staff performed onsite investigations to review infection control practices and collaborated with ACH staff to conduct extensive chart reviews of patients with *Bcc* infections and evaluate for common healthcare exposures. Findings were communicated to CDC who coordinated sharing of information between states. During March of 2018, PADOH issued a statewide Health Advisory encouraging the reporting of clusters associated with the product, and CDPH issued a call for cases through the CDPH infection preventionists listserv. While *Bcc* is not a reportable disease in either Pennsylvania or California, these states require reporting of unusual occurrences to health departments, such as clusters of infections. CDC also issued a request for cases through the Epidemic Information Exchange (Epi-X) to enhance case finding efforts.

When a state or local health department reported additional cases to CDC, a standard line list template was provided to the health department to gather case information such as site of infection, culture sources and dates, and potential product exposure, including lot numbers. Potential exposures were determined based on which lots the facility reported were in use at the facility during the patient's hospitalisation. This potential exposure information was utilised in the determination of case status.

A confirmed case was defined as a symptomatic patient who yielded a *Bcc*-positive culture first collected on or after 1 November 2017, matching or closely related (three or fewer pulsed-field gel electrophoresis (PFGE) band pattern differences) to an outbreak strain by PFGE. A probable case was a patient who yielded a *Bcc*-positive culture first collected on or after 1 November 2017 with unknown or pending strain type and had received care at a healthcare facility utilising an aqueous product from a lot manufactured by Manufacturer A contaminated with *Bcc*. A possible case was defined as a patient who yielded a *Bcc*-positive culture first collected on or after 1 November 2017 with unknown strain type and received care at a healthcare facility utilising aqueous product manufactured by Manufacturer A, but *Bcc* contamination was unknown (i.e. a different product or a NRCFP lot different from those that yielded *Bcc* on culture).

### Laboratory investigation

Early in the investigation, the Pennsylvania ACH that reported the initial cluster of infections performed cultures on 32 products that were commonly used on or by the cases in the ACH. The ACH also sent seven clinical isolates to the *Burkholderia cepacia* Research Laboratory and Repository at the University of Michigan [7]. PADOH, the New Jersey Department of Health (NJDH) and LACPH collected NRCFP samples for analysis from ACHs in their jurisdictions. Clinical isolates, including those from the initial cases, and product isolates were analysed by CDC laboratories. CDC received and analysed seven lots (each of which included three to six sub-lots) of the NRCFP using a modification of US Pharmacopeia (USP) <61> Microbiological Examination of Nonsterile Products: Microbial Enumeration Tests [8]. Product from ACHs tested by CDC was from unused bottles but were from opened boxes of product, while FDA tested product collected directly from Manufacturer A or from product that had remained in sealed, unopened boxes at the ACHs. Some lots were tested by both CDC and FDA laboratories. Initial species identification of all organisms was performed using MALDI-TOF with analysis using the Microbe.net database. CDC performed PFGE on *Bcc* isolates using a modified *Yersinia pestis* PulseNet protocol [9]. Chromosomal DNA from each isolate was digested with the *SpeI* enzyme, and gels run with 2 and 50 s pulse intervals on a linear ramp with a 21–22 h run time [9].

FDA collected a total of 14 NRCFP samples, from healthcare facilities in Maine (3), Pennsylvania (3) and New Jersey (4), and from two medical centres in California (4). A sample consisted of 10–12, four or eight oz unopened bottles of the NRCFP per lot of product. The product and environmental samples collected by FDA were analysed by FDA laboratories using standard methods, as outlined in the Bacteriological Analytical Manual for cosmetics and USP methods for drugs [5]. All *Bcc* isolates recovered by FDA were analysed using PFGE [5] and the above CDC run parameters to ensure comparable results between agencies. Isolates with three or fewer PFGE band pattern differences were considered part of the outbreak cluster [10].

### Traceback investigation

Local, state and federal health agencies conducted a traceback investigation to determine the manufacturing site of the NRCFP. Lot codes of all product samples collected for laboratory analysis at the healthcare settings were used to guide the investigation and determine the scope of the contamination.

### Environmental investigation

In March 2018, FDA inspected Manufacturer A to collect records and product, water and environmental samples. During this inspection, the investigators collected environmental samples consisting of 51 swabs, one water sample consisting of 16 subsamples, one sample of 10 4-oz containers of the NRCFP and multiple samples (different lots) of another hydrating cleanser product manufactured in the same facility. The investigators collected corresponding batch records, water sample records and cleaning and sanitising records with each sample collected.

## Results

### Epidemiologic investigation

**Fig. 1.**
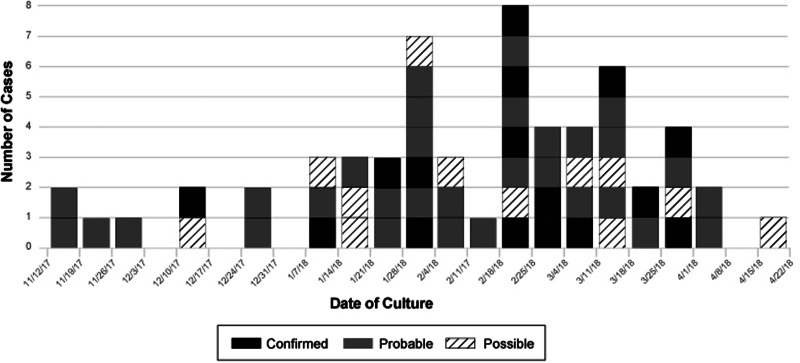
Confirmed, probable and possible clinical cases of *B. cenocepacia*, by date of illness onset for whom information was reported as of 27 May 2018 (*n* = 59).

As of 27 May 2018, there were 16 confirmed case-patients in three states: California (8), Pennsylvania (7), New Jersey (1); 31 probable case-patients in four states: California (21), Maine (2), Ohio (5), Pennsylvania (3); and 13 possible case-patients in three states: California (4), Nevada (4), New Jersey (5) (Fig. 1). Culture dates and sites of positive cultures were available for 59 of 60 cases. The available culture dates ranged from 16 November 2017 to 31 March 2018. Sites of positive cultures included urinary 35 (59%), respiratory 10 (17%), wound 4 (7%), blood 3 (5%), pelvic or peritoneal fluid 3 (5%), vaginal 1 (2%), urine and respiratory 2 (3%) and urine and wound 1 (2%). Two deaths were reported anecdotally in California; however, death data were not collected systematically, and it is unknown if the *Bcc* infection contributed to these deaths.

### Laboratory investigation

**Fig. 2.**
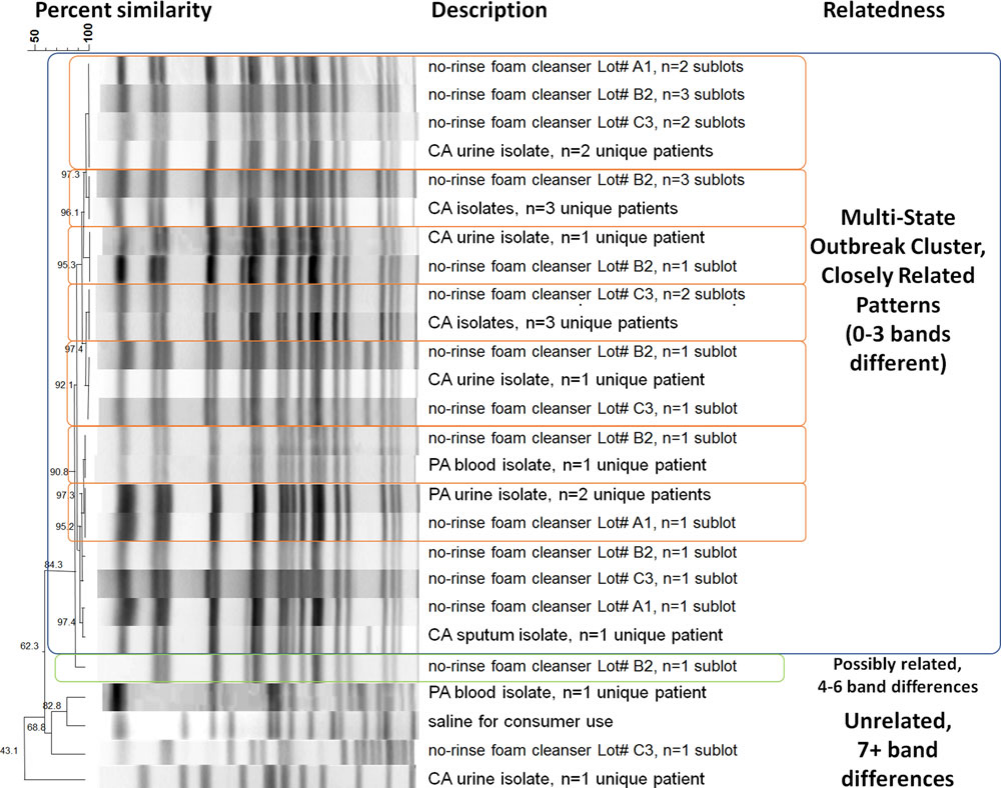
Dendrogram of *B. cenocepacia* isolate PFGE patterns as performed by CDC and FDA. Isolates of each unique band pattern were selected to represent patients' state of residence and product lot numbers. *n* values indicate the number of unique product batches and case patients. Patterns that are indistinguishable are marked with orange outlines. The outbreak cluster is indicated by the blue outline. The green outline indicates that the isolate is possibly related to the outbreak strain by a 4–6 band difference.

PADOH laboratory found eight of 15 bottles yielded *Bcc*. Pennsylvania clinical isolates were identified as *B. cenocepacia*, a species within *Bcc*. *B. cenocepacia* was cultured from multiple samples of three different lots of the NRCFP collected from ACHs and a sample collected by FDA directly from Manufacturer A [11]. LACPH laboratory cultured *B. cenocepacia* and *Pseudomonas monteilli* from an unopened bottle of NRCFP collected at the ACH. Further species identification of clinical case isolates and product isolates identified *B. cenocepacia*. The samples collected by NJDH for analysis did not yield *Bcc.* Clinical isolates from the 15 confirmed patients were found to be either indistinguishable or closely related to each other and to the product isolates by PFGE (Fig. 2). NRCFP collected and cultured by FDA and product sent directly to CDC for culture yielded *B. cenocepacia* isolates indistinguishable from clinical isolates by PFGE. Isolates from each of the three contaminated lots of NRCFP were indistinguishable from at least one clinical isolate. In addition to *B. cenocepacia* contamination in finished product, CDC and state partners identified contamination with *Pseudomonas monteilli*, *Burkholderia vietnamiensis* and *Pseudomonas putida*. *B. cenocepacia* counts ranged from not detected to 3.7 × 10^5^ CFU/ml from different bottles of NRCFP within the same lot.

### Traceback investigation

Traceback identified the contract manufacturer, Manufacturer A, for the three lots that yielded *B. cenocepacia.* Based on the shipping records, product labels, product lot codes obtained from the ACHs and discussions with the firms involved, FDA identified the supply chain of the lots that yielded *B. cenocepacia* isolates. This NRCFP had a fast turnaround time at the healthcare facilities and stock rotation may not have always used the oldest stocked product first (i.e. first in, first out rotation). Unlike medications, the lot number for these products is not routinely recorded in the patient record. Therefore, the exact lots of product that case-patients were exposed to could not be determined. Tested and traced product was available for sampling but may not have been the only product lots used on case-patients. The traceback and commercial relationships between the suspected product and firms were as follows: the NRCFP that case-patients were exposed to was produced by Manufacturer A, who was the exclusive contract manufacturer for Company A.

### Environmental investigation

During the on-site inspection at Manufacturer A by FDA and state investigators, poor manufacturing practices were observed for cosmetics and inadequate current good manufacturing practices (CGMPs) for drugs being manufactured. The firm shared manufacturing lines and equipment between cosmetic and drug products. Investigators observed liquid dripping from a crack on the bottom side of a jacketed tank (a heated vessel used to compound product) located in the compounding area, as well as liquid leaking from the outlet port on the bottom side of a jacketed tank in another compounding area. Vents above the jacketed tanks were blackened and dirty and black debris was observed on palletised raw material, which was directly below the vent. The ventilation system above the area designated for the weighing of components prior to formulation (pre-weigh booths) was broken, while heavy build-up of dust, debris and live insects were identified inside the pre-weigh booths and the staging area in front of the pre-weigh booths, which held opened exposed bags of raw materials. There was inadequate frequency of cleaning to prevent the build-up of dust and debris, and a live bird was observed in the raw material warehouse facility, while doors were kept open at the loading dock area. There were chipped and missing pieces of the conveyer belt on a product filler line, as well as wastewater flooding in the compounding area due to a non-functional floor drain. Worn and frayed water hoses, used for production, cleaning and sanitation operations, were submerged in wastewater on pitted and cracked floors. Finally, there were no handwashing stations throughout the production facilities [12].

According to FDA review of manufacturer logs, Manufacturer A had a flawed purified water system, and an adequate internal follow-up investigation was not performed when testing revealed microbiological contamination. Several water samples and cleansing foam product samples analysed by Manufacturer A had microbial growth too numerous to count on test samples that did not meet the firm's specifications, yet finished product was released for distribution, and later linked to illnesses. One environmental sample collected from a manufacturing line of the NRCFP yielded *Bcc* isolates; however, these isolates did not match the clinical isolates by PFGE. A variety of microorganisms, including pathogenic organisms, were identified from the samples collected from the environment, purified water system and finished products at the manufacturer (Table 1).

**Table 1.** Microbiological testing results from product and environmental samples collected by FDA from Manufacturer A and tested by FDA laboratories

Sample description	Number of positive samples	Microorganism isolates recovered
Water used for manufacturing	12	*Bacillus simplex*
		*Bacillus megaterium*
		*Brevundimonas diminuta/vesicularis*
		*Cronobacter sakazaki group*
		*Delfia acidovorans*
		*Pseudomonas putida*
		*Pseudomonas aeruginosa*
		*Klebsiella pneumoniae*
		*Kocuria rhizophila*
		*Mycobacterium mucogenicum*
		*Stenotrophomonas maltophilia, Sphingomonas*
		*paucimobilis*
		*Ralstonia pickettii*
Environmental samples	11	*Rhodotorula glutinis/mucilaginosa*
		*Bacillus subtilius/amyloliquefaciens/atrophaeus*
		*Bacillus boroniphilus*
		*Bacillus cereus/thuringiensis/mycoides*
		*Bacillus lichenformis*
		*Brevundimonas diminuta/vesicularis*
		*Burkholderia cepacia*
		*Enterobacter cloacae complex*
		*Comamonas testosteroni*
		*Ochrobactum anthropic*
		*Bacillus pumilus*
		*Solibacillus silvestris*
		*Paenibacillus lautus*
		*Pseudomonas stutzeri*
		*Staphylococcus cohni ssp urealyticus*
NRCFP	Four different production lots	*Burkholderia cepacia*
		*Pseudomonas* spp.
		*Staphylococcus* spp.
		*Staphylococcus epidermidis*
		*Neisseria* spp.
		*Bacillus* spp.
		*Streptococcus* spp.

### Product recalls and regulatory activities

On 23 March 2018, FDA and CDC provided representatives of Manufacturer A and Company A with updates on the outbreak investigation, and on 28 March 2018, Company A initiated a recall of the three lots of the NRCFP linked to the products used at the healthcare facilities where cases were exposed. This first recall included NRCFPs manufactured in August and September 2017. On 8 May 2018, Company A expanded the recall to include additional cosmetic products manufactured on the same production line as the NRCFP due to a potential risk of bacterial contamination. The second and expanded recall on 8 May 2018 addressed products manufactured on the same production line as the NRCFP from 1 August to 24 October 2017. Finally, on 24 May 2018, Company A recalled all over-the-counter drugs produced by Manufacturer A and still within their expiration date. On 6 February 2019, FDA issued a Warning Letter to Manufacturer A citing significant violations of the Food, Drug, and Cosmetic Act (FD&C Act) found during the follow-up investigation conducted as a result of this outbreak [12, 13].

### Public and provider communications

**Fig. 3.**
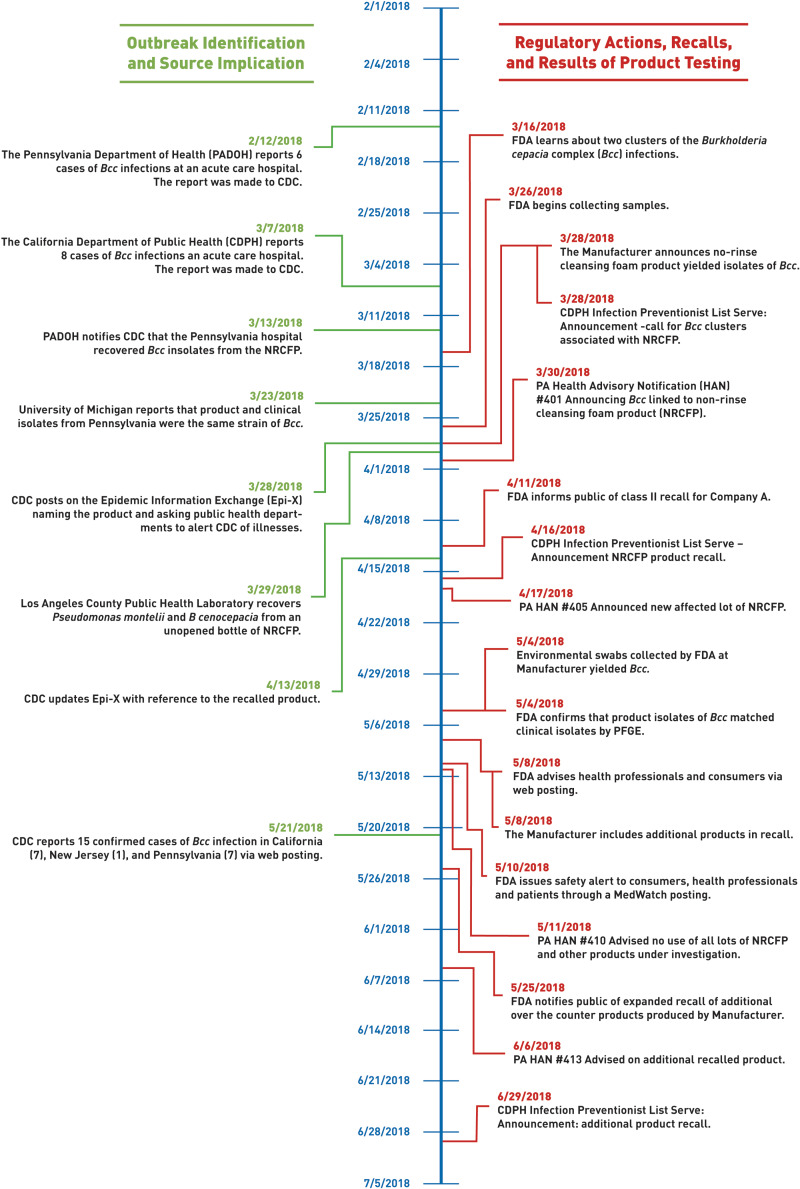
Timeline of events that took place including the outbreak identification, source implication, regulatory actions, recalls and results of product testing.

FDA, CDC and state and local partners issued notifications and health advisories throughout the duration of the outbreak response to inform the public and healthcare professionals of the outcomes of the investigation, including recall information, and provided specific advice to protect public health (Fig. 3) [11, 13–15]. FDA and CDC shared specific guidance advising patients, pharmacies, ACHs and other healthcare facilities and companies that purchased the recalled product to: (1) immediately quarantine material under their control and contact the manufacturer; (2) immediately stop using and dispensing the recalled product; and (3) follow the recall instructions for the recalled lots and avoid the use of all other lots of the product while further investigation was being conducted.

## Discussion

Following timely reports by the ACH infection prevention and control professionals of clusters of cases and of the clinical laboratory results (which implicated the NRCFP) FDA, CDC and state and local partners successfully confirmed NRCFP as the source of the outbreak of *B. cenocepacia* infections based on epidemiologic and laboratory evidence. The identification and removal of the contaminated product from the market was crucial in stopping this outbreak and preventing further illnesses. Exposure information collected by the Pennsylvania ACH and PADOH was a critical first step that guided the investigation. State and local partners collected product samples for analysis that resulted in the isolation of the outbreak strain of *B. cenocepacia*. FDA's laboratory and environmental investigations further confirmed the source of the outbreak as the NRCFP. The investigation at Manufacturer A revealed significant lapses in CGMPs and quality management throughout the production area, which likely led to this contamination. Public health communications by federal partners informed health professionals to stop using the product. Finally, the firm recalled the contaminated product from the market. Subsequent actions by all agencies involved, including the firm, removed the contaminated products from the market and prevented further infections.

This outbreak investigation highlights the continued risk for *Bcc* infections in healthcare settings due to the contamination of medical products, provides further evidence of *Bcc* as an infectious risk to patients and highlights the importance of ongoing surveillance for *Bcc* infections in ACH settings. Although not currently a reportable disease, frontline staff followed requirements to report suspect clusters of infections to public health, triggering the identification of, and quick response to, this outbreak.

This investigation also demonstrated that *Bcc* can cause a range of clinical infections even with exposure to the same contaminated product. The most common site of infection was the urinary tract, as the product is primarily used for perineal care but is also often applied liberally over the skin on other parts of the body, which likely led to the positive wound cultures. Contact between contaminated skin and the mouth and nose or inhalation could have possibly led to the positive sputum cultures. Among the initial six cases identified in Pennsylvania, only one had a urine culture positive for *B. cenocepacia*. Conversely, the majority of the cases from the initial facility in California had positive urine cultures, an unusual site for *Bcc* infections. Thus, at the time CDPH reported the California cluster it was not clear that the clusters were related. Continued investigation by PADOH and the Pennsylvania ACH led to the identification of four more cases, all with positive urine cultures, which helped establish the connection between the outbreaks. Communication across public health agencies further helped confirm that the product identified in Pennsylvania was also used at the facility in California.

One limitation of the investigation was that because it was not documented in the medical record which lot of the NRCFP was used on an individual patient, we were unable to connect cases directly to exposure to a specific lot. We relied on reports from the ACH of which lots were used at the ACH during patient hospitalisation. Tested and traced product represented product available for sampling and may not have been the only product lots used on case-patients. Reports of *Bcc* infections were submitted on a voluntary basis by healthcare providers and microbiology laboratories, and it is likely that not all cases were identified. Clinical information was collected at the time of the report and did not always include the final disposition of the patient; therefore, it is not known whether deaths of case patients were directly or indirectly attributed to the outbreak-associated *B. cenocepacia* infections. A further limitation of this investigation was that cases were defined by positive culture results and case definitions did not include any additional clinical criteria as this information was not collected systematically for all cases.

*Bcc* are considered environmental, opportunistic pathogens that frequently contaminate pharmaceuticals and cosmetics and researchers have suggested that they be considered for inclusion in regular surveillance to monitor environmental contamination at production facilities [16, 17]. The ubiquitous occurrence of *Bcc* in water, which is a primary component in the manufacture of many medical devices, drugs and cosmetic products, and the resistance of *Bcc* to many disinfectants and preservatives, has led to multiple outbreaks of *Bcc* involving intrinsic contamination of these non-sterile products [5, 6]. The first line of defence against contamination of finished products by pathogenic microorganisms, such as *Bcc*, is the establishment and robust implementation of carefully designed and controlled CGMP operations. Manufacturers are responsible for assessing relevant production factors, including the characteristics of each raw material, sanitary equipment design, the processing steps and the capability of the finished product to support microbial proliferation. Manufacturers should also consider the intended use, the route of administration and the risk factors of the population who will use the product. Manufacturers who make non-sterile water-based products should have a heightened sense of awareness of the possibility of contamination in the component water from deficient water systems and should take measures to prevent the release of contaminated product. Manufacturers should monitor water quality, proactively ensure sanitary design, control bioburden levels, prevent objectionable contamination and establish appropriate microbiological quality standards for the finished product [18]. Any of the described CGMP lapses could have led to the contamination of the NRCFP, which resulted in multiple infections of hospitalised patients. The response of the firm to their discovery of microbial contamination in the product was inadequate and resulted in the shipment of contaminated product. Manufacturer A's practices resulted in extensive recalls and an FDA-issued Warning Letter to the firm.

*Bcc* causes disease primarily in immunocompromised populations, but may also cause illness in non-immunocompromised, previously healthy individuals [19]. The case-patients infected in this outbreak were recovering from surgeries and medical conditions that may have made them susceptible to infection. It is also important to note that the NRCFP was not intended to be rinsed off of the skin, which may have increased the likelihood of acquiring an infection, especially among hospitalised patients who may have been immunocompromised or critically ill [14].

The NRCFP met the cosmetic product definition under section 201(i) of the FD&C Act because the product is intended to be used for ‘cleansing, beautifying, promoting attractiveness, or altering the appearance’. However, it was intended to be used by people who are unable to bathe or shower because of physical or health limitations, including immuno-compromised patients in a healthcare setting. The combination of the intended use for vulnerable populations, intrinsically contaminated product linked to an outbreak and poor manufacturing practices precipitated the NRCFP recall. It also led to a subsequent recall, by Manufacturer A, of additional drug and cosmetic products, including shampoo, conditioners, hair styling products and beard creams that were beyond the product identified as the source of the outbreak. Failure to recover microbial growth in other products does not indicate the products were free of contamination because microbial contamination is a stochastic occurrence and is generally not evenly distributed on equipment or in product batches. When objectionable microbial contamination is detected in a facility or product, it is typically an indicator of underlying problems, such as lack of CGMPs. Since a single test is just a snapshot in time, relying solely on testing is inadequate, though a properly designed and robust testing protocol can help a manufacturer identify contamination issues. Manufacturers should ensure the entire manufacturing process operates in accordance with CGMPs. With the firm's use of shared manufacturing equipment and the firm's inadequate CGMPs, there were likely additional instances of contamination that were not detected via FDA's and the firm's limited testing.

## Conclusions

FDA, CDC, state and local health agencies collaborated successfully leading to the identification and removal of a contaminated patient skin care product from the market to prevent additional cases, protect public health and end the outbreak. Furthermore, the collaborating partners produced evidence that enabled FDA to issue a Warning Letter to Manufacturer A. This outbreak investigation's findings echoed previous *Bcc* outbreak investigations, emphasising the importance of sanitary practices and CGMPs, as well as, most importantly, taking appropriate actions in response to contamination discovered by the firm. This company did not adhere to manufacturing practices that ensured cosmetic products were manufactured and controlled in accordance with appropriate safety and quality standards. Their failure to adhere to adequate manufacturing and safety protocols increased the risk of healthcare-associated infections and other adverse events in product users.

## Data Availability

Data supporting the findings of this outbreak are noted in this manuscript.

## References

[1] Depoorter E (2016) *Burkholderia*: an update on taxonomy and biotechnological potential as antibiotic producers. Applied Microbiology and Biotechnology 100, 5215–5229. 27115756 10.1007/s00253-016-7520-x

[2] Sommerstein R (2017) *Burkholderia stabilis* outbreak associated with contaminated commercially-available washing gloves, Switzerland, May 2015 to August 2016. Euro Surveillance 22. 10.2807/1560-7917.ES.2017.22.49.17-00213 PMC5727593 29233255

[3] Jones AM, Dodd ME and Webb AK (2001) *Burkholderia cepacia*: current clinical issues, environmental controversies and ethical dilemmas. European Respiratory Journal 17, 295–301. 11334134 10.1183/09031936.01.17202950

[4] Glowicz J (2018) A multistate investigation of health care-associated *Burkholderia cepacia* complex infections related to liquid docusate sodium contamination, January-October 2016. American Journal of Infection Control 46, 649–655. 29329922 10.1016/j.ajic.2017.11.018 PMC6192668

[5] Becker SL (2018) Outbreak of *Burkholderia cepacia* complex infections associated with contaminated octenidine mouthwash solution, Germany, August to September 2018. Euro Surveillance 23. 10.2807/1560-7917.ES.2018.23.42.1800540 PMC6199865 30352639

[6] Coenye T (2002) Comparative assessment of genotyping methods for epidemiologic study of *Burkholderia cepacia* genomovar III. Journal of Clinical Microbiology 40, 3300–3307. 12202570 10.1128/JCM.40.9.3300-3307.2002 PMC130787

[7] United States Pharmacopeial Convention (2012) Microbiological examination of nonsterile products: microbial enumeration tests (general information Chapter 61). In The United States pharmacopeia tr, and The national formulary (ed.), United States Pharmacopeial Convention. Rockville, MD, USA: United States Pharmacopeial Convention.

[8] U.S. Centers for Disease Control and Prevention (2006) One-Day (24–28h) Standardized Laboratory Protocol for Molecular Subtyping of Yersinia pestis by Pulsed Field Gel Electrophoresis (PFGE). U.S. Centers for Disease Control and Prevention. Available at https://pulsenetinternational.org/assets/PulseNet/uploads/pfge/yersinia_Apr2006.pdf.

[9] Barrett TJ, Gerner-Smidt P and Swaminathan B (2006) Interpretation of pulsed-field gel electrophoresis patterns in foodborne disease investigations and surveillance. Foodborne Pathogens and Disease 3, 20–31. 16602976 10.1089/fpd.2006.3.20

[10] U.S. Food and Drug Administration (2018) Outbreak investigation of *B. cepacia* complex linked to medline remedy essentials no-rinse cleansing foam. Available at https://www.fda.gov/food/outbreaks-foodborne-illness/outbreak-investigation-b-cepacia-complex-linked-medline-remedy-essentials-no-rinse-cleansing-foam.

[11] U.S. Food and Drug Administration (2019) Warning letter: shadow holdings DBA Bocchi Labs. Available at https://www.fda.gov/inspections-compliance-enforcement-and-criminal-investigations/warning-letters/shadow-holdings-dba-bocchi-labs-561273-02062019.

[12] Government of Canada (2018) Expanded Recall: Medline Canada, Corp. Recalls Various Medline Cosmetic Products. Government of Canada. Available at https://www.healthycanadians.gc.ca/recall-alert-rappel-avis/hc-sc/2018/66910r-eng.php.

[13] U.S. Centers for Disease Control and Prevention (2010) *Burkholderia cepacia* in healthcare settings. Available at https://www.cdc.gov/hai/organisms/bcepacia.html.

[14] U.S. Food and Drug Administration (2018) Product recalls. Available at https://www.accessdata.fda.gov/scripts/ires/index.cfm?Product=162968.

[15] Wong SCY (2020) Polyclonal *Burkholderia cepacia* complex outbreak in peritoneal dialysis patients caused by contaminated aqueous chlorhexidine. Emerging Infectious Diseases 26, 1987–1997. 32818396 10.3201/eid2609.191746 PMC7454066

[16] Tavares M (2020) *Burkholderia cepacia* complex bacteria: a feared contamination risk in water-based pharmaceutical products. Clinical Microbiology Reviews 33. 10.1128/CMR.00139-19 PMC7194853 32295766

[17] U.S. Food and Drug Administration (2017) FDA Advises Drug Manufacturers that Burkholderia cepacia Complex Poses a Contamination Risk in Non-Sterile, Water-Based Drug Products. U.S. Food and Drug Administration. Available at https://www.fda.gov/drugs/drug-safetyand-availability/fda-advises-drug-manufacturers-burkholderia-cepacia-complex-poses-contamination-risk-non-sterile.

[18] Torbeck L (2011) *Burkholderia cepacia*: this decision is overdue. PDA Journal of Pharmaceutical Science and Technology 65, 535–543. 22293841 10.5731/pdajpst.2011.00793

[19] Tavares Mariana (2020) *Burkholderia cepacia* Complex Bacteria: a Feared Contamination Risk in Water-Based Pharmaceutical Products. Clinical Microbiology Reviews 33(3). 10.1128/CMR.00139-19. PMC7194853 32295766

